# Burnout and psychiatric morbidity among medical students entering clinical training: a three year prospective questionnaire and interview-based study

**DOI:** 10.1186/1472-6920-7-6

**Published:** 2007-04-12

**Authors:** Marie E Dahlin, Bo Runeson

**Affiliations:** 1 Department of Clinical Neuroscience, Division of Psychiatry St. Göran, Karolinska Institutet, 112 81 Stockholm, Sweden

## Abstract

**Background:**

Mental distress among medical students is often reported. Burnout has not been studied frequently and studies using interviewer-rated diagnoses as outcomes are rarely employed. The objective of this prospective study of medical students was to examine clinically significant psychiatric morbidity and burnout at 3^rd ^year of medical school, considering personality and study conditions measured at 1^st ^year.

**Methods:**

Questionnaires were sent to 127 first year medical students who were then followed-up at 3^rd ^year of medical school. Eighty-one of 3^rd ^year respondents participated in a diagnostic interview. Personality (HP5-i) and Performance-based self-esteem (PBSE-scale) were assessed at first year, Study conditions (HESI), Burnout (OLBI), Depression (MDI) at 1^st ^and 3^rd ^years. Diagnostic interviews (MINI) were used at 3^rd ^year to assess psychiatric morbidity. High and low burnout at 3^rd ^year was defined by cluster analysis. Logistic regressions were used to identify predictors of high burnout and psychiatric morbidity, controlling for gender.

**Results:**

98 (77%) responded on both occasions, 80 (63%) of these were interviewed. High burnout was predicted by Impulsivity trait, Depressive symptoms at 1^st ^year and Financial concerns at 1^st ^year. When controlling for 3^rd ^year study conditions, Impulsivity and concurrent Workload remained. Of the interviewed sample 21 (27%) had a psychiatric diagnosis, 6 of whom had sought help. Unadjusted analyses showed that psychiatric morbidity was predicted by high Performance-based self-esteem, Disengagement and Depression at 1^st ^year, only the later remained significant in the adjusted analysis.

**Conclusion:**

Psychiatric morbidity is common in medical students but few seek help. Burnout has individual as well as environmental explanations and to avoid it, organisational as well as individual interventions may be needed. Early signs of depressive symptoms in medical students may be important to address. Students should be encouraged to seek help and adequate facilities should be available.

## Background

Repeated evidence that medical students are subjected to considerable stress has been published over the last decades [1-4]. There is evidence that mental distress during medical school predicts later problems in physicians [5,6], which in addition to the personal suffering of the individual doctor might negatively affect patient care [7]. It is known that physicians do not seek the kind of professional help for themselves as they would provide for their patients [8-11]. Medical students seem to adopt a similar behaviour [12,13]. However, we know little about the prevalence of clinically significant mental distress. Most studies of medical student's distress rely on questionnaire data or qualitative studies from e.g. focus group interviews. There are, to our knowledge, few studies that present data based on diagnostic interviews on either medical students or physicians. Prevalence rates for medical students vary; from interview-based studies 6–8% one-year prevalence of depression in an American sample was reported twenty years ago [14] and more recently in a UK sample, psychiatric morbidity was found in 16% of medical students [15]. Based on self-ratings, prevalence rates of depression in the range of 14–24% (BDI scores) have been reported [16-18], and psychiatric caseness in 22–36% (GHQ-12) in a longitudinal sample [15]. In a study of Swedish university students, 14–19% reported having been depressed during the previous academic year [19]. Among senior UK physicians, 29% were considered likely cases of anxiety (HAD-A) and 11% of general practitioners considered likely cases of depression (HAD-D) [20]. In an Italian study of hospital physicians and general practitioners, the prevalence of psychiatric morbidity was found to be 22.3% (GHQ-12) [21].

Retrieving knowledge about presence of psychiatric conditions is important in itself and if found, would need attention. In addition it might indicate a vulnerability that medical school could not be held responsible for. While burnout or stress-reactions are conceived of as reactions to a pressing environment, psychiatric disorders such as bipolar disorder, depression, anxiety are considered inherently multi-factorial, with e.g. hereditary contributions. Medical students have been found to have higher rates of depression than the general population even before entering medical school [14].

Whether the demands of medical education or dispositional factors of the individuals choosing this career has the greatest explanatory power has sometimes been addressed [22-24]. Burnout, which is perceived as denoting particular work-related adverse reactions, may be considered a measure of distress induced from studies. It has, however, also proven to be associated with neuroticism and Type A behaviour [25]. Further, performance-based self esteem (PBSE) has been suggested as a predictor or even a prerequisite for burnout [26]. Cross-sectional data in a medical student sample revealed high levels of PBSE and gave support for a close relationship between PBSE and burnout dimensions Exhaustion and Disengagement [27]. There are some reports on burnout in medical students; Guthrie found low levels in British medical students [15], while Dyrbye, using less conservative criteria, defined as many as 45% of North American medical students in a multicenter study as burnt out [4].

There has been a trend among medical schools around the world to develop curricula with integration of preclinical and clinical training from an early stage of the education. The present study was undertaken at the Karolinska Institutet medical school, which offers a "traditional" curriculum with initial preclinical studies, later followed by clinical courses. Such traditional curricula have been associated with more negative attitudes towards medical school among students compared to integrated curricula [28], but there is no evidence that any model would attract more vulnerable students. The transition from preclinical to clinical training has been identified as a crucial stage of medical school, regarding student stress [29]. This is also when students in these "traditional" medical schools begin to grasp an image of their future profession.

The objective of this study was to determine the presence of clinically significant psychiatric morbidity at the initial stage of clinical training and to examine its relation to burnout levels. In addition we wanted to explore the effects of personality traits and the study environment for these outcomes.

## Methods

### Participants and procedures

All students (N = 127, 57.5% women, mean age 23.8/sd 4.7 years) entering medical school at Karolinska Institutet in the fall 2001 were sent a postal questionnaire, 113 (89.0%) responded. A follow-up questionnaire was sent as they reached their third curricular year (spring 2004 – autumn 2005), with a response rate of 102 (80.3%). Ninety-eight (77.2%) students responded on both occasions. Fifteen (11.8%) students were not eligible for follow-up (not registered at third curricular year) during the study period. Thirteen of these were reached by telephone interviews, revealing that 6 had left medical school, 4 had moved to continue their medical education at a different university and 3 had not yet reached their third year.

Respondents at third year (n = 102) were asked to participate in an interview. Of these, 81 students were interviewed and 80 (78.4% of approached, 63% of the original cohort) had returned completed questionnaires on both occasions. Interviews were held by physicians trained in psychiatry. Interviewers had joint training on the interview manual, but no inter-rater reliability tests were performed. The mean interval between response and interview was 6 weeks (range 0–26 six weeks, mode 2 weeks).

Written informed consent was obtained for the questionnaires and for the interviews, respectively. Students identified with clinically significant suffering were given counselling on appropriate help. Approval of the study was obtained by the Ethics Committee at Karolinska Institutet.

### Measures

The questionnaire contained items on previous and concurrent contact with a professional for psychological or personal problems (3^rd ^year) and whether students had a paid employment outside medical school during semesters (1^st ^and 3^rd ^years).

We used two factors from HP5-i (Health Relevant Personality from a five factor perspective inventory) [30] as personality measures. The HP5-i comprises 20 items, measuring five aspects of personality, each aspect being a facet of one of the "Big Fives". Impulsivity (Cronbach's α 0.81, a facet of Conscientiousness) and Negative Affectivity (Cronbach's α 0.62 a facet of Neuroticism) were included in the present study. Each factor consists of four items, scored 1–4, from which the mean is drawn.

Study conditions were monitored by three factors from the Higher Education Stress Inventory, HESI [31]. Each item is rated on a four-point Likert scale, 1–4. Scores are computed as means of pertaining items, where high values are undesirable. Seven factors have been derived from the HESI in a cross-sectional study of a larger sample of medical students [31]. We used the following in this study: *Worries about future endurance/competence *(Cronbach's α 0.72), 3 items, e.g. "I worry about long working hours and responsibilities in my future career"; *Workload *(Cronbach's α 0.62), 3 items, e.g. "The pace of studies is too high" and "The literature is too difficult and extensive"; *Financial concerns *(Cronbach's α 0.65), 2 items, e.g. "As a student, my financial situation is a worry" and "I am worried about my future economy and my ability to repay student loans". Due to low internal consistency of other study condition factors, these were not used.

Burnout was assessed by the student version of the Oldenburg Burnout Inventory, OLBI, at baseline and follow up [27,32]. The OLBI is an inventory designed for assessing burnout regardless of occupation. It includes two dimensions; Exhaustion (Cronbach's α 0.81) and Disengagement (Cronbach's α 0.74) from studies, each containing eight positively or negatively worded statements, scored 1–4, from which the mean is drawn.

Performance-based self-esteem was assessed by the PBSE scale, comprising four items rated on a 5 point Likert scale [26]. Cronbach's α was 0.72.

Self-rated depression was assessed by a slightly modified version [31] of the Major Depression Inventory [33], with four response alternatives instead of the original six. A sum score for all ten items, Depressive symptom load, is given. This variable had a minimum score (no symptoms) of 0 and a maximum of 30 points. Cronbach's α was 0.84.

Psychiatric diagnoses were set by the Swedish version of the Mini International Neuropsychiatric Interview (MINI) 5.0.0, yielding DSM-IV diagnoses and recommended for epidemiological studies [34]. We did not include the modules of psychotic disorders, drug abuse or antisocial personality disorder. Psychiatric morbidity (caseness) was defined as having a diagnosis of Major Depression, Dysthymia, a history of hypomania and/or mania, Social phobia, Generalised anxiety disorder, Alcohol abuse, Alcohol dependency or Bulimia nervosa.

### Statistics

The SPSS version 14.0 was used for statistical analyses. Paired samples t-tests were used for changes over time. Intra-class correlations were computed for Study conditions, Exhaustion, Disengagement and Depressive symptom load. We used k-means cluster analysis to identify two subgroups of students according to 3^rd ^year exhaustion and disengagement, since no cut off levels for OLBI subscales are available. Differences between proportions were compared by Chi^2 ^tests. Determinants of psychiatric morbidity and burnout were examined by logistic regressions.

## Results

From first to third year, Worries about future endurance/capacity and Financial concerns increased, while Workload decreased significantly. Neither burnout measures Exhaustion and Disengagement, nor Depressive symptom-load changed over time (table 1). There were no gender differences in any of the measures at first year. At third year, female students had higher scores on Worries about future endurance/capacity, 2.84 (s.d. 0.70) than male students, 2.44 (s.d. 0.72), p = 0.00.

**Table 1:** Personality, performance-based self-esteem at 1^st ^year. Study conditions, Burnout, Depression and Alcohol consumption at 1^st ^and 3^rd ^years. N = 98

		**1^st ^year**		**3^rd ^year**		**p**	**Intra-class corr**
Category (Measure)		Mean	SD	Mean	SD		
Personality (HP5-i)	Impulsivity	2.15	0.63	-	-		
	Negative Affectivity	2.11	0.58	-	-		

PBSS	Performance-based self-esteem	3.27	0.89				

Study Conditions s (HESI)	I Worries about future endurance/competence	2.44	0.76	2.70	0.74	.001	.593***
	IV Workload	2.46	0.61	2.29	0.57	.023	.319***
	VII Financial concerns	2.22	0.89	2.42	0.84	.013	.618***

Burnout (OLBI)	Exhaustion	2.32	0.53	2.40	0.50	.120	.488***
	Disengagement	1.91	0.45	1.95	0.49	.502	.311***

(MDI)	Depressive symptom load	7.96	5.23	7.78	5.19	.083	.484***

***p < 0.001, paired samples t-test

Non-responders at third year (n = 15) had higher levels than responders at both occasions on first year Disengagement (2.23 (s.d. 0.54) vs. 1.93 (s.d. 0.47), p = 0.017), Exhaustion (2.65 (s.d. 0.53) vs. 2.36 (s.d. 0.52), p = 0.037) and Workload (2.78 (s.d. 0.56) vs. 2.38 (s.d. 0.59), p = 0.012).

### Classification of subgroups according to third year burnout

A two-cluster analysis identified a high burnout subgroup, with high levels of both variables, (n = 46; 47%) and a low-burnout (low on both, n = 52; 53%) subgroup. Since the K-means cluster analysis is sensitive to ordering of cases in data file, several analyses were run with different ordering; all yielded the same solution. Exhaustion mean in the high burnout group was 2.76 (s.d. 0.34) vs. 2.08 (s.d. 0.39) among low burnout, and Disengagement was 2.31 (s.d. 0.41) vs. 1.62 (s.d. 0.28).

### Prediction and explanation of burnout at third year

Logistic regressions were performed with burnout as dependent variable (high = 1, low = 0) and personality, first year distress and study conditions from first and third year as independent variables. Crude and adjusted OR's are displayed in table 2. The unadjusted analyses showed that Impulsivity, Depressive symptom load, Disengagement, Exhaustion, Worries about future endurance/capacity and Financial concerns measured at first year were all risk factors for third year burnout, as were study conditions measured at third year (Worries..., Workload and Financial concerns). An unadjusted model of Impulsivity as only dependent, had Cox & Snell R^2 ^0.055 and Nagelkerke R^2 ^0.075. In the adjusted model with significant first year measures as dependent variables, only Impulsivity and Financial concerns reached statistical significance. In a second step, we entered significant predictors from the adjusted model with third year study conditions, controlling for gender. Impulsivity was still significant, as was 3^rd ^year Workload.

**Table 2:** Factors explaining burnout at third year of medical school. N = 98.

	Crude			Model 1			Model 2		
	OR	95% CI	p	Adj OR	95% CI	p	Adj OR	95% CI	p
Gender (woman = 1)	1.27	0.56–2.89	.568	1.14	0.45–2.93	.779	0.98	0.37–2.64	.976
Negative Affectivity	1.63	0.81–3.31	.174						
Impulsivity	2.29	1.13–4.63	.021	2.21	1.03–4.77	.042	2.88	1.23–6.74	.015
Performance-based self-esteem	1.04	0.66–1.63	.870						
Depressive symptom load 1^st ^yr	1.12	1.03–1.22	.011	1.01	0.88–1.15	.904			
Disengagement 1^st ^yr	3.04	1.17–7.89	.022	1.74	0.46–6.64	.415			
Exhaustion 1^st ^yr	3.57	1.50–8.50	.004	1.71	0.50–5.87	.398			
Worries about future endurance/competence 1^st ^yr	1.76	1.02–3.06	.044	1.12	0.57–2.19	.738			
Workload 1^st ^yr	1.47	0.75–2.86	.260						
Financial concerns 1^st ^yr	1.88	1.16–3.05	.011	1.84	1.02–3.31	.042	1.25	0.62–2.52	.535

Worries about future endurance/competence 3^rd ^yr	2.64	1.42–4.89	.002				1.85	0.88–3.93	.107
Workload 3^rd ^yr	5.69	2.24–14.44	.000				3.95	1.37–11.35	.011
Financial concerns 3^rd ^yr	2.28	1.33–3.89	.003				1.62	0.76–3.44	.210

Logistic regressions. Model 1 includes all significant explanatory variables from 1^st ^year, method enter. Cox and Snell R^2 ^0.18, Nagelkerke R^2 ^0.23.

Model 2 includes the significant predictive variables from model 1 (controlling for gender) and third year study conditions, method enter. Cox and Snell R^2 ^0.28, Nagelkerke R^2 ^0.37.

Since Financial concerns was significant, we examined if the groups differed in the rates of paid employment during semesters. Among high burnouts, 21.7% (10) worked during semesters at 1^st ^year and 54.3% (25) at 3^rd ^year, while among low burnout the corresponding numbers were 30.8% (16) at 1^st ^year and 57.7% (30) at 3^rd ^year, not significant between groups at any stage.

### Interviews

Table 3 shows the results from the diagnostic interviews (n = 81) students. In total, 27% (n = 21) were assessed to fulfil criteria for at least one diagnosis. Among these, 3 (14%) had an ongoing contact with a professional due to perceived psychological problems and 6 (29%) had sought help at some point during the course of medical school. Comorbidity was present in four students; two had social phobia and generalised anxiety disorder, one was diagnosed with social phobia and alcohol abuse and one student had alcohol dependency and a history of mania. There were no gender differences, except for alcohol abuse, which was only detected in men. The distribution of psychiatric morbidity over burnout subgroups is also shown (n = 80); high-burnout individuals reported a history of depression more often than did subjects with low burnout. The correlation between burnout category and psychiatric morbidity was 0.177 (Kendall's tau_b, p = 0.116).

**Table 3:** Interviewer-rated psychiatric conditions (MINI)

	All (n = 81)		Low burnout (n = 46)		High burnout (n = 34)	
	n	(%)	n	(%)	n	(%)
Major depression	5	(6.2)	1	(2.2)	4	(11.8)
*History of depression (n = 76)*	*32*	*(41.6)*	*13*	*(28.9)*	*18*	(58.1*)
Dysthymia	1	(1.3)	0	(0)	1	(2.9)
*Hypomania ever*	*4*	*(4.9)*	*0*	*(0)*	*3*	*(8.8)*
*Mania ever*	*1*	*(1.2)*	*0*	*(0)*	*1*	*(2.9)*
Social Phobia	6	(7.6)	2	(4.3)	4	(11.8)
Generalised Anxiety Disorder	3	(3.7)	2	(4.3)	1	(2.9)
Obsessive Compulsive Disorder	0	(0)	-	-	-	-
Panic disorder	0	(0)	-	-	-	-
Post Traumatic Stress Disorder	0	(0)	-	-	-	-
Alcohol abuse	5	(6.2)	2	(4.3)	3	(8.8)
Alcohol dependence	6	(7.4)	2	(4.3)	4	(11.8)
Bulimia	1	(1.2)	1	(2.2)	0	(0)
Anorexia nervosa	0	(0)	-	-	-	-

Psychiatric caseness†	21	(27.2)	9	(19.6)	12	(35.3)

Variables and numbers in italics refer to historic, not present symptoms.

†Presence of either Major Depression, Dysthymia, Hypomania ever, Mania ever, Social phobia, Generalised Anxiety disorder, Alcohol abuse, Alcohol dependency or Bulimia nervosa.

* p > 0.05, Fisher's Exact test

### Prediction of psychiatric morbidity

We examined possible predictors of psychiatric morbidity by another set of logistic regressions (table 4). The unadjusted analyses showed that high levels of Performance-based self-esteem, Depressive symptom load and Disengagement, measured at first year, increased the risk of having a psychiatric diagnosis three years later. In the adjusted model, containing those univariately significant dependent variables, Depressive symptom load alone remained significant.

**Table 4:** Variables predicting clinically significant psychiatric morbidity in third year undergraduate medical students, controlling for gender. (N = 80)

Predictors/explanatory variables	Crude OR	95% CI	p	Adj. OR	95% CI	p
Gender (woman = 1)	0.50	0.18–1.38	.181	0.32	0.10–1.03	.056

Impulsivity	2.01	0.82–4.92	.127			
Negative affectivity	1.66	0.68–4.06	.265			
PBSE	1.93	1.01–3.37	.045	1.48	0.74–2.98	.266

Disengagement 1^st ^year	3.64	1.08–12.26	.037	1.13	0.24–5.36	.874
Exhaustion 1^st ^year	2.23	0.80–6.17	.124			
Depressive symptom load	1.16	1.04–1.30	.008	1.17	1.02–1.35	.028

Worries about future endurance/competence 1^st ^year	1.84	0.92–3.68	.084			
Workload 1^st ^year	2.00	0.88–4.54	.096			
Financial concerns 1^st ^yr	1.54	0.87–2.72	.141			

Logistic regression. Adjusted model by method enter of variables with significant crude associations, Cox & Snell R^2 ^0.16, Nagelkerke R^2 ^0.23

## Discussion

The major findings of this longitudinal study of medical students were that about 25% of the interviewed medical students had a clinically significant psychiatric diagnosis and that higher levels of the personality trait Impulsivity predicted burnout, even when concurrent Workload was controlled for.

### Mental distress, burnout and psychiatric morbidity?

Burnout is traditionally viewed as a reactive pattern towards negative working conditions, affecting vitality and emotions towards the work. In the present study, high and low burnout subgroups were identified by cluster analysis, thus reflecting a distribution within the sample studied; thus high burnout should not be perceived as a measure of clinically significant distress. Hence, there were no statistically significant differences between the two groups regarding current psychiatric morbidity. However, a history of depression was more common among high-burnout individuals, which might reflect the same vulnerability as was suggested from the unadjusted analyses, where high levels of 1^st ^year depressive symptom load was associated with high burnout.

### Burnout

First year ratings of depressive symptoms, disengagement, exhaustion and Worries about future capacity, were all univariately significant predictors, but not in the adjusted model. The latter had moderate correlations with all the others mentioned, which were internally strongly (r > 0.6) correlated. Thus the unique effect of each is blurred in the controlled model, and cannot be separated out. They may still all be relevant. High burnout was predicted by trait Impulsivity, thought to be associated with high risk-taking and unhealthy behaviour [30]. Among personality traits, Neuroticism (or negative affectivity) and Type A behaviour have shown associations with burnout [25]. High impulsivity may be associated with a shortage of strategic planning and goal-directedness towards the studies, which may put the impulsive individual at risk in a long-term, demanding education. Findings from a 12-year longitudinal study of medical graduates in the UK showed that Conscientiousness (being the negatively oriented factor of Impulsivity) had negative correlations with burnout and was predictive of a "surface-disorganised" approach to work [24]. Impulsivity may also indicate "impatience". During clinical training, students often spend considerable time without specific assignments, waiting for a physician/teacher to come to the ward to teach and supervise. Students' wish to learn and to be of use might then be frustrated and, high impulsivity students might in particular, tend to react with a loss of engagement. Our results confirm previous studies suggesting that personality do have an impact on burnout. However, the variance explained by personality was only 5–7%. Other variables in our model, as well as intra- and extracurricular stressors not assessed here, such as life events, are probably important [4]. The design of this study will not allow further conclusions regarding the controversy on whether personality or environmental factors as major causes of distress, though [24,35].

The finding that financial concerns at first year predicted burnout could not be explained by a greater extent of paid employment outside medical school among high burnout individuals. Financial concerns and debts have been shown to be associated with emotional exhaustion in physicians and mental distress in medical students [36,37]. Concurrent workload was, as expected, a significant explanatory variable, although the confidence interval was wide and the strength of the association thus quite inexact, as is of course also the direction of causality. Exhaustion is perceived as related to job demands [32], where workload is one important aspect.

### Psychiatric morbidity, help-seeking and determinants

Considering the limited number of interviewed students and their proportion of the original sample (n = 81, 63%), we do not present the numbers as estimates of prevalence. Still, an ongoing psychiatric condition in over 25% of the interviewed students is more than most other studies report. An earlier version of the MINI has been shown to be slightly over-inclusive for generalised anxiety and give false-positive cases on mania [38], which may have affected our results. All interviewed students were fully active in their studies, indicating that their functional level was not very heavily affected, and the recorded states probably of mild to moderate severity. The composite outcome is different from e.g. depression only, for which the level in our study, 6%, was similar to that of a study two decades ago [14]. Our findings were lower that those of a recent Brazilian study using the Self-Report Questionnaire-20 (SHR-20), where 40.2% of medical students were reported to have a common mental disorder [39]. Only a small proportion of those identified as cases had an ongoing treatment of any kind, resembling findings of a recent American study [16]. The Students' health services at Karolinska Institutet provide a low-threshold consulting facility with nurses, social workers and possibility to see a psychiatrist. We lack information on whether those who actually had sought help had used this channel or others outside medical school, or on students' knowledge about its existence. The reluctance among medical students as well as physicians to seek help is well acknowledged [9-13] and is an important target for actions. Besides active teaching on the subject of help-seeking in medical school, the social norms among medical students and physicians on this subject should be more openly debated. To lower thresholds for seeking help, specific treatment programs for a medical students and health personnel may also be beneficial.

Psychiatric morbidity is a divergent entity, but although it lacks in theoretical rigour, it allows for an attempt to find risk measures for distress of *clinical *significance. The unadjusted and the adjusted results differed. Disengagement and Depressive symptom load at first year predicted psychiatric morbidity at third year in the unadjusted analyses, as did performance-based self-esteem. Only depressive symptoms reached significance in the adjusted model. As stated above, the dependent variables are inter-correlated, and may still be relevant. Previous mental health problems, intensity personality trait, perceived medical school stress and the wishful thinking coping style were all significant predictors of self-reported mental health problems in need of treatment among physicians in their fourth postgraduate year [6]. We think that signs of depression among first year students should be paid attention to, and that they might serve as an indication to take preventive action against future distress. We found high levels of previous depressive episodes, also shown in American students [14], and it has been found that personality explains stress and burnout [24]. This may lead to suggestions that admission processes should assess e.g. mental health history in applicants to medical school, in order to select less vulnerable individuals. Without evidence that medical students with psychiatric morbidity either perform worse or drop out, such conclusions should not be drawn. While severe depressive states, hypomania, mania and substance abuse may deteriorate performance, mild depressions, anxiety or eating disorders cannot be concluded to do so. Further, Firth-Cozens showed that physicians with higher depression scores were more empathic than their peers, which may well be an attractive feature [40]. People do differ, even medical students, and we should strive for educational and work environments that can harbour people with different strengths and vulnerabilities, and maybe help guide students into career paths that are optimal for them also from a health perspective.

A higher level of performance-based self-esteem increased the risk of psychiatric morbidity in the unadjusted regression analysis, but in contrast to earlier cross-sectional findings it was not associated with burnout [27]. The lack of longitudinal associations may be due to the interval in time in itself; PBSE levels may be subjected to major changes in young adults, attending a formative education, and less stable than reported from larger and less selected samples [26].

### Limitations

Non-responders at follow-up differed from the sample included in this study by having higher exhaustion, disengagement and workload scores at baseline. Thus, those students who experienced most stress at first year were lost, which probably has influenced the results, at least regarding burnout. It would have been desirable to include a measure of social support in medical school in the multivariable models; regrettably, the HESI factor Non-supportive climate had to be left out due to low internal consistency. While gender stratification in the multivariable analyses would have been valuable, the study had not enough power for that.

## Conclusion

A major strength of the present study is the interview-based, diagnostic procedure, showing that a substantial proportion of medical students had an ongoing psychiatric condition, and very few of these had sought professional help. Actions should be taken to encourage medical students to seek help for psychological problems and to provide adequate facilities. Interventions addressing the mental health of medical students might be directed towards those revealing depressive symptoms already during their first year of medical school. Further research is needed to confirm our findings, and intervention studies are called for. Individual as well as organisational interventions should be targeted to prevent burnout among medical students.

## Competing interests

The author(s) declare that they have no competing interests.

## Authors' contributions

MD planned the study, collected the data, performed statistical analyses and interpretation of data and drafted the manuscript. BR conceived of the design of the study, arranged funding, supervised, participated in the interpretation of data and helped draft the manuscript. Both authors have read and approved of the final manuscript.

## Pre-publication history

The pre-publication history for this paper can be accessed here:


